# Hypertensive Response to Exercise in Athletes: Unremarkable Finding or Relevant Marker for Future Cardiovascular Complications?

**DOI:** 10.1155/2022/8476751

**Published:** 2022-11-01

**Authors:** Laura Würzburger, Patrick Wiech, Valentina A. Rossi, Daniel Neunhäuserer, Stefano Caselli, Christian M. Schmied, David Niederseer

**Affiliations:** ^1^Department of Cardiology, University Hospital Zurich, Zurich, Switzerland; ^2^Sport and Exercise Medicine Division, Department of Medicine, University of Padova, Italy; ^3^Cardiovascular Center Zurich, Hirslanden, Klinik im Park, Zurich, Switzerland

## Abstract

**Background:**

In the general population, hypertensive response to exercise (HRE) predicts new-onset resting hypertension or other cardiovascular diseases.

**Methods:**

PubMed was searched for English articles published between January 1st 2000 and April 30th 2020. Additional studies were identified via reference lists of included studies. 92 papers were selected for full text analysis, finally 30 studies were included.

**Results:**

The results from 5 follow-up studies suggested an association between HRE and the risk of developing hypertension, while 10 studies reported a link with adverse cardiovascular events in the general population. Another study showed an association between HRE and future hypertension in athletes after a follow-up of 7 years. HRE in athletes was associated with left ventricular hypertrophy in three studies. Two other studies showed a link between HRE and focal myocardial fibrosis in triathletes and myocardial injury, respectively. One study found lower Apoliprotein-1 serum levels in athletes with HRE leading to a higher risk for cardiovascular disease. Only in one study no association with cardiovascular dysfunction in athletes with HRE was found.

**Conclusions:**

Based on current evidence, HRE is not a normal finding in athletes. If detected, it should be interpreted as a risk factor for future cardiovascular complications. Future research should address the adequate follow-up and management of athletes with HRE.

## 1. Introduction

Overwhelming evidence shows that regular aerobic exercise is favorable for the cardiovascular system [1]. More specifically, several studies have shown that physical activity including aerobic and resistance exercise lowers both systolic and diastolic blood pressure [2–4]. This effect is particularly strong in people with high resting blood pressure [3, 5]. Moreover, exercise reduces the usual cardiovascular risk factors such as hypercholesterolemia, overweight/obesity, and type 2 diabetes (by reducing insulin resistance) [5].

As within the normal population, hypertension is the most common cardiovascular disease in athletes. Conditions typically associated with hypertension in athletes are high body mass index, positive family history, and unhealthy lifestyle. Other common factors include intake of anti-inflammatory drugs, dietary supplements, abuse of anabolic steroids, and psychosocial pressure [6]. However, also the type of sport may play a minor role [7].

To increase the cardiac output, the systolic blood pressure physiologically raises linearly with the increment of load via increased sympathetic tone. Thus, the metabolic needs of the working muscles can be covered. The diastolic blood pressure remains stable or decreases minimally [8].

To assess the effect of physical workload on blood pressure regulation, the type of workload should be considered. In terms of different sports disciplines, dynamic sports showed higher systolic blood pressure (SBP) values due to high workloads, whereas static sports showed higher diastolic blood pressure (DBP) values as a result of increased peripheral resistance [7].

It has been observed that some individuals respond to physical stress with an exaggerated increase in systolic blood pressure (SBP) and diastolic blood pressure (DBP). Such a hypertensive response to exercise (HRE) has been also found in subjects without any previously known cardiovascular disease and may predict new-onset resting hypertension and/or future cardiovascular disease [8–11].

Currently, there is no consensus on how to define the blood pressure limits for HRE. The European Society of Cardiology (ESC) guidelines report reference values for SBP of ≥210 mmHg for men and SBP of ≥190 mmHg for women [12]. Caselli et al. reported slightly higher values of SBP, i.e., 220/85 mmHg for males and 200/80 mmHg for females [13].

The pathophysiology of HRE is poorly understood. Several mechanisms related to endothelial dysfunction and vascular stiffness have been demonstrated. Both may limit vasodilatation in response to exercise, leading to HRE. Endothelial dysfunction mainly results in HRE in younger individuals, whereas increased arterial stiffness is more likely to be responsible for inadequate vasodilation during exercise in older individuals [11].

Since sedentary subjects with HRE have a higher risk of developing cardiovascular events, it has been questioned whether HRE in athletes should be interpreted as a normal adaptation to training, or whether it represents an imbalance between cardiac output and peripheral vascular resistance, and could therefore have a potential future clinical impact on hypertension, cardiovascular events, and mortality [14].

Here, we review the current knowledge of HRE and its possible clinical consequences with the focus on athletes. We hypothesize that HRE in athletes is a risk factor for future cardiovascular complications, due to several training hours a day with repetitively high blood pressure loads.

Further insights on previous literature have been presented in the online supplement.

## 2. Methods

The following study designs were considered in this systematic review: prospective or retrospective cohort, cross-sectional, and case-control. Studies that reported exercise induced blood pressure response and evaluated the clinical implications of HRE in the athletic and non-athletic population aged >18 years were included. Studies in English were considered. Papers analyzing HRE in a non-athletic population with previous diseases were excluded.

The software EndNote was used for reference management.

### 2.1. Information Sources and Searches

A systematic literature review was performed according to the procedure specified by the preferred reporting items for systematic reviews and meta-analyses (PRISMA) guidelines [15]. For literature extraction, papers were searched within the PubMed database using following search terms: “exaggerated exercise blood pressure,” “exercise-induced hypertension,” “exercise-related blood pressure,” and “hypertensive response to exercise in athletes.” Animal and in-vitro studies were excluded. Papers published and those in publication process (i.e., in press and available in PubMed) between January 1st 2000 and April 30th 2020 were considered. In addition, references from identified papers and suggestions for similar articles were hand-searched, screened, and analyzed.

Duplicates were systematically examined and removed. Relevant articles were identified by screening the title and reading the abstract. For the final eligibility, full-text analysis was performed. Data extraction was performed for each eligible study by one independent author.

Information was extracted from each included study regarding the following aspects: characteristics of participants including gender, age, athletic condition, blood pressure response to exercise and observations associated with long-term outcomes such as hypertension, cardiovascular events, or cardiac remodeling.

**Figure 1:**
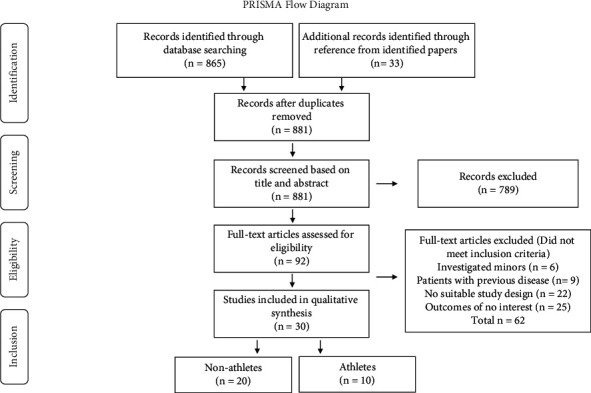
PRISMA flow diagram: this figure shows the PRISMA flow diagram of identified papers. The citations yielded by the electronic search were screened by reviewing the title or abstract of each. 789 papers were excluded, and 92 publications were assessed for eligibility. From these, 62 did not suit the inclusion criteria and were excluded. Finally, 30 articles were included in the qualitative synthesis.

The literature search yielded a total of 881 papers. Of these, 17 were excluded after checking for duplicates and 789 after screening for title and abstract. Among the 92 articles selected for full-text analysis, 62 did not meet the inclusion criteria. In conclusion, 30 papers were considered relevant, among these, 10 were suitable to address the question whether HRE in athletes represents a risk factor for future cardiovascular complications (Figure 1).

### 2.2. Patient and Public Involvements

No patients were involved in this study.

## 3. Results

Characteristics of the included studies are described in Tables 1 and 2.

From 30 included studies, 20 analysed HRE in non-athletes and 10 in athletes. The calculated median age of non-athletes was 48.75 years, and the interquartile range (IQR) was 10. The median percentage of men was 69, and the IQR was 44.15. The calculated median age of the athletes was 38.95 years, and the IQR was 19. The median percentage of male athletes was 86.5, and the IQR was 35.

Five prospective studies found a link between HRE and the risk of developing hypertension in non-athletes, while just one prospective study showed that athletes with HRE had a higher risk of developing arterial hypertension. 10 studies showed a relationship between cardiovascular events in non-athletes with HRE. In three different studies, an association between HRE in athletes and LV hypertrophy was found. Two studies reported a relationship between HRE in triathletes and myocardial injury with focal myocardial fibrosis. A cross-sectional study found lower Apoliprotein-A1 levels in athletes with HRE, suggesting a higher risk for cardiovascular disease. Only one study could not find any link between HRE in athletes and cardiovascular dysfunction.

### 3.1. Results of Individual Studies

#### 3.1.1. HRE as a Risk Factor for Hypertension in Non-Athletes

Berger et al. showed in a large cohort of 7082 subjects that the probability of developing new-onset hypertension during a follow-up of 5 years was increased in individuals with HRE as compared to those without HRE [23]. Farah et al. reported outcomes of a small cohort of individuals with HRE, where 84% (11 out of 13) developed hypertension after two years [24]. In over 20 years, Yzaguirre et al. found that a DBP of more than 95 mmHg at peak or a SBP more than 180 mmHg during moderate exercise is associated with a 70% probability of developing hypertension [32]. The study by Sharabi et al. showed that individuals with HRE had almost a six-fold risk of developing hypertension after a mean follow-up of 5.7 years, while no significant differences were found with respect to the risk of developing other cardiovascular conditions [34]. Ito et al. monitored middle-aged men over 10 years. Moreover, these authors observed a positive correlation between an HRE and the development of a new-onset hypertension [26]. Miyai et al. examined 239 Japanese middle-aged men with high-normal blood pressure and followed up with them for six years. In a cox multivariate analysis of risk factors, including HRE, familiarity for hypertension, and weight gain as risk factors for incidence of hypertension, HRE was found to predict the risk of developing hypertension [30].

Schultz et al. examined 75 individuals with HRE of whom 56% had masked hypertension, showing a direct association [19]. Similarly, Kayrak et al. found a prevalence of 41% of masked hypertension in 61 patients with HRE, [17] and Sharman et al. found a prevalence of 58% for masked hypertension in 72 subjects with HRE [18]. Since masked hypertension is associated with increased mortality but cannot be diagnosed during office blood pressure measurement, Schultz et al. speculated that masked hypertension could be detected during low-intensity exercise testing as HRE has high specificity and is more time-efficient than a 24 h ambulatory blood pressure monitoring [19]. In a more recent study, Schultz et al. found an association between elevated SBP during early stages of exercise stress test and hypertension that was only identified by 24 h ambulatory blood pressure monitoring [20].

#### 3.1.2. HRE and Risk for Cardiovascular Events

Several studies reported increased cardiovascular events and mortality in subjects with HRE at moderate exercise compared to individuals without HRE. After a follow-up of 15 years Hietanen et al. found that an elevated ankle blood pressure in combination with elevated blood pressure levels during exercise was a significant independent predictor of coronary heart disease in subjects with normal resting blood pressure [25]. Kjeldsen et al. showed that an elevated SBP during moderate workload predicts cardiovascular mortality independently from other known cardiovascular risk factors, while no association was found for SBP at peak exercise and cardiovascular death [27]. Over a mean follow-up of 10 years, Kurl et al. reported an increased risk for stroke in patients with a high SBP rise per minute of exercise [28]. Weiss et al. found that elevated blood pressure at rest, at low-intensity exercise, and at maximal exercise independently predicts future cardiovascular death [31]. Laukkanen et al. demonstrated that an excessive SBP rise in response to exercise is associated to an increased risk of acute myocardial infarction [29]. Lewis et al. followed-up a subgroup of 3045 individuals with HRE from the Framingham Heart Study over 20 years and showed that HRE was associated with an increased risk for future cardiovascular disease beside resting blood pressure and other conventional risk factors [9].

Mottram et al. compared echocardiographic findings in patients with HRE with and without resting hypertension with subjects with normotensive response to exercise and no history of hypertension: they report an association between HRE and subtle systolic dysfunction in HRE, while no correlation between HRE and left ventricular hypertrophy was observed [33]. Left ventricular diastolic dysfunction and exercise intolerance have also been observed in patients with HRE [21]. Using speckle tracking imaging, Yang et al. demonstrated longitudinal myocardial dysfunction among subjects with HRE. However, conventional echocardiographic examination did not show differences in diastolic parameters, the left ventricular mass index, or ejection fraction between individuals with and without HRE [22]. Chung et al. found an association between female sex, arterial stiffness, advanced diastolic dysfunction, and HRE. Even after adjustment for other risk factors, the association remained significant. Differences were found according to sex and age [16].

#### 3.1.3. HRE in Athletes

Blood pressure response to exercise in athletes differs from non-athletes, as athletes present higher maximal exertion blood pressure values compared to non-athletes and, thus, could represent a physiological adaptation mechanism to higher loads [43].

Currie et al. showed that athletes with HRE did not have an impaired sympathetic reactivity, left ventricular dysfunction, or increased arterial stiffness in contrast to untrained individuals with HRE and concluded that HRE in athletes is a compensatory mechanism to meet the higher demands of peripheral blood flow rather than as a dysfunction of the cardiovascular system [35]. Caselli et al. showed that the body mass index, resting SBP, and the type of training (i.e., endurance exercise) predict HRE in athletes [13]. The same group showed that normotensive young athletes with HRE had an increased incidence (hazard ratio 3.6) of resting hypertension at 7 years of follow-up as compared to athletes without HRE (HRE: 13.5% vs. no HRE: 3.5%) [14]. During follow-up, no major adverse cardiovascular events and no significant cardiac remodeling was documented [14]. Leischik et al., documented left ventricular hypertrophy and left atrial enlargement potentially caused by HRE in 51 triathletes and speculated that HRE might play a role in sudden cardiac death, training-induced cardiac fatigue, and atrial fibrillation [37]. A significant and dose-dependent relationship of left ventricular remodeling (myocardial thickening and higher left ventricular mass) to HRE was documented. Tahir et al. showed a dose-dependent higher incidence of focal myocardial fibrosis in competitive male triathletes with HRE [39]. Another study of the same group examined 30 master triathletes with and without myocardial fibrosis after an endurance race and showed lower left atrial ejection fraction, more elevated NT-proBNP values, and HRE in those athletes with myocardial fibrosis. The authors speculated that HRE might have contributed to cardiac injury and myocardial fibrosis [40]. Bauer et al. investigated the SBP/metabolic equivalent of task slope in elite athletes and its relation to the BP response to a maximum exercise test in elite athletes and their performance. The study showed that athletes with the lowest SBP/metabolic equivalent of task slope exhibit the lowest maximum SBP and achieved the highest absolute and relative workload, implying a better performance [41]. Kim et al. found an increase in left ventricular mass index, diastolic dysfunction, and elevated pulse pressure in marathon runners with HRE, compared to sedentary subjects with early-stage hypertension, with a positive correlation between elevated pulse pressure and diastolic dysfunction [42]. Malek et al. analyzed 30 male long-term ultra-marathon runners: athletes with high-normal resting blood pressure had an increased interventricular septal thickness and higher left and right ventricular mass index; those findings were even more accentuated in subjects with high-normal BP and HRE. Athletes with isolated HRE did not show left ventricular hypertrophy [38]. Turmel et al. examined 44 endurance athletes (with and without HRE) using exercise testing, blood sampling during exercise and recovery, 24 h ambulatory blood pressure monitoring, and 24 h Holter electro cardiography monitoring. Athletes with HRE had lower levels of Apolipoprotein-A1 and showed higher SBP levels during 24 h blood pressure monitoring compared to athletes without HRE. As Apolipoprotein-A1 seems to be cardioprotective and a reduction in Apolipoprotein-A1 correlates with a higher risk for cardiovascular disease, this might be a sign of the detrimental effects of HRE in athletes on the cardiovascular system [36].

## 4. Discussion

In this systematic review, we collected current knowledge to answer the question whether HRE in athletes is associated with the onset of cardiovascular diseases. In summary, this is a hypothesis-generating review supporting the idea that HRE is associated with future development of hypertension and/or cardiovascular events in non-athletes and that in athletes may be associated with mild cardiac structural or functional abnormalities as well as future hypertension.

If a link could actually be demonstrated in further prospective studies, HRE would be an early and easily detectable marker in the long-term development of arterial hypertension, and other cardiovascular events—as exercise testing is routinely performed in athletes and would therefore have enormous prognostic meaning.

### 4.1. HRE in Athletes Is Associated with Cardiovascular Implications in the Future

Caselli et al. found an association between HRE in athletes and new-onset hypertension. Over a follow-up period of almost 7 years, they did not report any major cardiovascular event nor significant changes in cardiac remodeling in athletes with HRE [26]. Therefore, the positive effects of exercise training and the consequently improved cardiovascular fitness likely outweigh the detrimental effects of HRE in athletes [14].

Caselli and Leischik report different findings regarding the myocardial alterations due to HRE. In the study by Caselli et al., athletes did not develop significant cardiac remodeling during follow-up. Leischik et al., however, showed a positive correlation between HRE and myocardial thickening [37]. These discrepant findings could be explained by the difference in intensity and frequency of the training: Leischik et al. examined triathletes competing for Ironman-distances, who had a much more vigorous training load than the athletes analyzed in the study by Caselli et al. Another reason is, that the population analyzed by Caselli et al. was young, and in 7 years of follow- up, significant events couldn't be expected.

Kim et al. showed that HRE is correlated with an increased LV mass index, diastolic dysfunction, and elevated pulse pressure in athletes. Differently, training-induced left ventricular hypertrophy in athletes is typically associated with normal cardiac function [42]. Tahir et al. observed that athletes with HRE were more likely to develop myocardial fibrosis [39]. These results provide further support to the hypothesis that HRE might cause myocardial injury and, therefore, cardiac dysfunction [40].

Turmel et al. observed higher SBP values of 24 h blood pressure monitoring as well as lower Apolipoprotein-A1 serum levels in athletes with HRE than in athletes with normal blood pressure response to exercise [36].

However, some studies do not support the evidence that HRE in athletes is associated with adverse cardiovascular events. Currie et al. assumed that HRE in athletes is a compensatory mechanism due to an increased peripheral blood flow under effort [35]. Malek et al. only found an association with LV hypertrophy when HRE was combined with high-normal blood pressure at rest, while HRE alone did not correlate with myocardial alterations and, as such, HRE in athletes may not be related to the onset of hypertension [38].

Most papers are cross-sectional studies and, as such, are not suitable to investigate any cause-effect relationship regarding adverse outcomes. Nevertheless, most of the authors speculate that the beneficial impact of exercise overcomes the negative effects of HRE in the long term. Overall, mostly men were analyzed in the included studies, so that a gender-related effect cannot be excluded and further studies are required to determine the HRE incidence and its consequences in females, as well.

The cause of HRE in athletes remains unclear at the moment. An endothelial dysfunction with lack of vasodilation leading to reduced peripheral resistance with consequent exaggerated blood pressure response could be an underlying mechanism, similar to non-athletes. At the same time, possible causes of hypertension such as the intake of antirheumatic drugs, adrenergic agonists, and other doping substances should be considered and excluded.

More research on this topic needs to be undertaken to understand the exact pathophysiology of HRE in athletes.

### 4.2. Clinical Relevance and Prognostic Value

Despite methodological differences such as threshold for HRE, time of onset of exercise-induced hypertension, and exercise testing protocol, the prognostic significance of HRE has been substantiated in several studies.

As mentioned in the introduction, a consensus on the definition of HRE does not exist. Since athletes are capable of higher exercise performance, compared with the normal population, and thus achieve higher blood pressure values at maximal exercise, different thresholds should be used to diagnose HRE. According to different studies, we suggest cut-off values of ≥210/110 mmHg for men and ≥190/95 mmHg for women from the non-athletic population and values of ≥220/85 mmHg, respectively, ≥200/80 mmHg for athletes [14].

**Figure 2:**
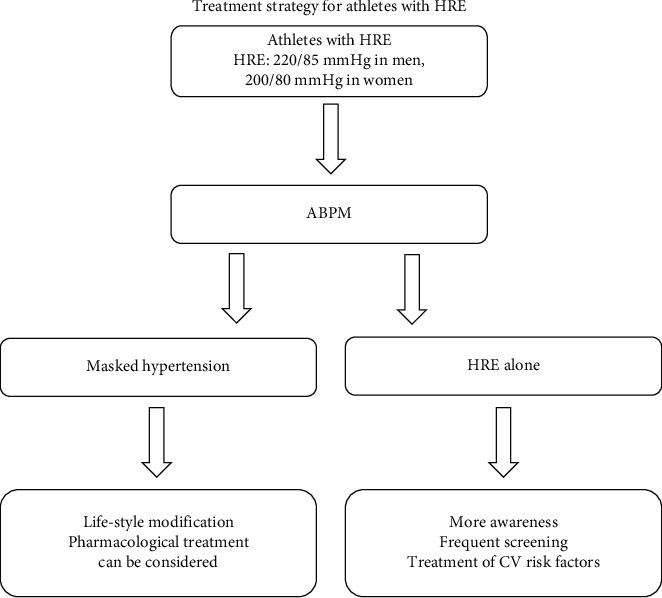
Diagnostic and therapeutic procedure for athletes with HRE. This figure shows a schematic treatment strategy for athletes with HRE. After the identification of HRE in athletes, an ambulant blood pressure monitoring (ABPM) should be performed to diagnose a possible masked hypertension. Depending on the findings of the ABPM, a suitable therapy can be applied.

Based on current evidence, we propose an algorithm for a diagnostic and therapeutic approach in athletes with HRE (Figure 2). If an athlete is diagnosed with HRE, he/she should undergo 24 h blood pressure monitoring to detect a possible masked hypertension, which should lead to medical therapy according to current guidelines [44]. If the athlete has an isolated HRE, he/she should be screened regularly and treated for cardiovascular risk factors.

It is still debated whether a medical therapy in athletes with HRE is reasonable or not. The aim of such a therapy would be to counteract HRE during training and competitions in order to limit the onset of aortic stiffness or left ventricular hypertrophy. In athletes with high-normal resting blood pressure, life-style modifications like weight reduction, alcohol and smoking restriction are recommended as first-line interventions by the current guidelines of the sports cardiology section of the European Association of Preventive Cardiology (EAPC). Antihypertensive drugs should be started in athletes presenting with resting SBP values ≥180 mmHg or DBP ≥110 mmHg and/or at high risk for cardiovascular complications [44]. A similar approach has been recommended for athletes with known essential hypertension, with no restriction to sport participation needed [6].

Since angiotensin II influences the vascular and myocardial response to exercise, angiotensin II receptor blocker (AT-I) or angiotensin converting enzyme inhibitor (ACE-I) could represent a first-line therapy in athletes with HRE. However, at the moment, a pharmacological therapy is not indicated in neither population, unless a masked hypertension is discovered, as no evidence is yet available that antihypertensive treatment in HRE is in any way beneficial [11]. It is important to recognize that these two drugs do not affect exercise capacity nor are they listed as doping, while other antihypertensives are considered as doping substances. For some skill sports like archery and shooting, beta-blockers for example, are considered as doping, while exercise performance might be negatively affected for endurance sports. Diuretics are forbidden in all kind of competitive sports [44]. However, both AT-I and ACE-I are contraindicated during pregnancy and female athletes should be aware of this.

LV hypertrophy caused by endurance training is reversible after a detraining period. Differently, cardiac remodeling triggered by hypertension usually responds well to antihypertensive medication, however, LV hypertrophy may not be reverted anymore, thus representing a rational to treat athletes with HRE [44].

Analogous, in non-athletes with HRE, a 24 h blood pressure monitoring should be sought to identify masked hypertension. Patients diagnosed with HRE should be allowed to perform high-intensity exercise even if they develop high SBP levels during exercise. Frequent physical activity with alternating workload intensity and lifestyle-modifications should be implemented to reduce the general risk of cardiovascular disease.

Further comments about the pathophysiology of HRE and the possible role of HRE as a precursor of cardiovascular diseases have been presented in the online supplement.

### 4.3. Limitations

We limited our search to PubMed and English articles and herewith smaller studies in journals not listed in PubMed or published in another language may have been missed. However, all major studies relevant to this topic are published in journals listed in PubMed. Furthermore, we only included studies in humans and excluded possibly interesting studies in animal models or in-vitro. Most studies included in this review are cross-sectional studies and consequently not suitable to identify causal relationships or predict outcomes. A significant disadvantage is the lack of information of the temporal sequence of the events investigated. A clear and universally accepted definition for HRE is needed, and standardized measurement methods should be proposed and implemented. Moreover, data extraction and screening were searched by only one researcher.

## 5. Conclusions

**Figure 3:**
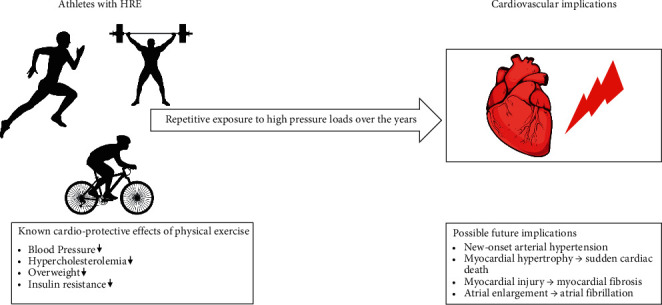
Scheme of possible future implications in athletes with hypertensive response to exercise. This figure illustrates athletes with hypertensive response to exercise and the possible future cardiovascular implications throughout the years. At the beginning, the cardio-protective effects of physical exercise seem to outweigh the negative risk factors associated with HRE. Due to the repetitive pressure overloads during the years, the negative effects could predominately outweigh the benefits.

The main goal of this narrative review was to summarize the actual knowledge about cardiovascular consequences of HRE in athletes. To date, there are several studies reporting an association between HRE in athletes and adverse cardiovascular events due to chronic exposure to pressure overload such as the onset of hypertension, adverse myocardial remodeling, or injury (Figure 3). However, HRE is not a normal finding, neither in the general population, nor in athletes. To establish a more casual relationship, further prospective studies are needed.

## Figures and Tables

**Table 1:** Study characteristics of included studies with non-athletes.

Author (year)	Study size (n)	Definition for HRE	Age (years)	Gender (% male)	Exercise testing protocol	Study design	Summary of findings
Chung et al. [16]	797	SBP ≥210 mmHg for male and ≥190 mmHg for female	64 ± 10	62	Symptom-limited supine bicycle testing; increase in workload of 25 W every 3 min	Cross-sectional	Subjects with HRE had higher LVMI and diastolic dysfunction. Arterial stiffness was related to HRE. Women had a higher prevalence of HRE
Kayrak et al. [17]	61	SBP ≥210 mmHg for male and ≥190 mmHg for female	47.5 ± 9.7	77	Bruce protocol	Cross-sectional	Masked hypertension prevalence is higher in patients with HRE and is related to higher BMI, adverse lipid profile, higher DBP during exercise and nocturnal DBP fall
Sharman et al. [18]	72	SBP ≥210 mmHg for male and ≥190 mmHg for female or DBP ≥105 mmHg in both	54 ± 9	60	Bruce protocol	Cross-sectional	Masked hypertension can be identified with 24 h ABPM and is highly prevalent in patients with HRE
Schultz et al. [19]	75	SBP ≥210 mmHg for male and ≥190 mmHg for female	54 ± 9	52	Cycle ergometer steady state heart rate at 60–70% of age-predicted maximal heart rate	Cross-sectional	56% of subjects with HRE had masked hypertension. During low-intensity exercise, brachial BP measurements were increased in subjects with MH. Light exercise BP predicts the presence of MH with high specificity
Schultz et al. [20]	100	SBP ≥150 mmHg at stage one of the test	56 ± 9	72	Bruce protocol	Cross-sectional	SBP ≥150 mmHg during early stages of exercise stress testing is associated with hypertension as identified through 24 hour ABPM
Takamura et al. [21]	129	SBP/DBP ≥210/105 mmHg in males, and ≥190/105 mmHg in females	63 ± 9	64	Bruce protocol	Cross-sectional	Subjects with HRE had an impaired LV diastolic function and exercise intolerance
Yang et al. [22]	171	SBP ≥200 mmHg for male and ≥190 mmHg for female	48 ± 8	56.7	Bruce protocol	Cross-sectional	Conventional echocardiographic examination showed no differences in LVMI or ejection fraction. Analysis with speckle tracking imaging demonstrated that individuals with HRE had impaired myocardial function
Berger et al. [23]	7082	Definition according to the values at peak exercise	48 ± 9	73	Bruce protocol	Prospective (5 ± 3 years)	14.6% developed new-onset hypertension
Farah et al. [24]	30	SBP ≥200 mmHg or DBP ≥100 mmHg	45 ± 10	53	Bruce protocol	Prospective (2 years)	84% of subjects with HRE developed hypertension during the following 2 years
Hietanen et al. [25]	3808	≥215 mmHg	50	66	Symptom-limited bicycle testing; increase in workload of 40–50 W every 3 min	Prospective (15 years)	In persons with normal resting blood pressure, elevated ankle blood pressure in combination with HRE, was a significant independent predictor for coronary heart disease
Ito et al. [26]	733	SBP ≥200 mmHg	41 ± 5	100	Stepwise graded exercise test protocol consisting of three grades—ergometer	Prospective (10 years)	HRE was positive correlated to hypertension at rest
Kjeldsen et al. [27]	1999	SBP ≥200 mmHg	40–59	100	Symptom-limited bicycle testing; increase in workload of 50 W every 6 min	Prospective (21 years)	Higher SBP values during moderate effort were related to CV mortality. The maximal SBP during exercise didn't show an influence on CV death
Kurl et al. [28]	1026	No definition	52.5 ± 4.7	100	Symptom-limited bicycle testing; increase in the workload of 20 W/min	Prospective (10.4 years)	A high SBP rise per minute of exercise was independently associated with an increased risk of stroke
Laukkanen et al. [29]	1731	No definition	52.3 ± 5.3	100	Symptom-limited bicycle testing; increase in the workload of 20 W/min	Prospective (12.7 years)	An exaggerated rise in SBP during exercise was related to an increased risk of acute myocardial infarction
Lewis et al. [9]	3045	No definition	43	47	Bruce protocol	Prospective (20 years)	HRE was associated with adverse cardiovascular events
Miyai et al. [30]	239	No definition	42.3 ± 5.9	100	Symptom-limited bicycle testing; a linear-slope method at a rate of 12.5 W × min^−1^ was used to increase workload	Prospective (5.1 years)	HRE and high-normal BP represent a risk factor for the development of hypertension
Weiss et al. [31]	6578	Bruce stage 2 SBP ≥180 mmHg	46	55	Bruce protocol	Prospective (20.1 ± 4 years)	Elevated BP at rest, at low-level exercise, and at maximal exercise were all associated with CVD death independently from non-BP risk factors
Yzaguirre et al. [32]	107	SBP/DBP ≥215/95 mmHg at maximum, SBP/DBP ≥180/90 mmHg at moderate exercise	25.7 ± 11.1	72	Symptom-limited bicycle testing; increase in the workload of 25 W/min	Prospective (20 years)	Subjects presenting DBP over 95 mmHg at peak exercise or SBP >180 mmHg during moderate exercise had a 70% risk of developing hypertension
Mottram et al. [33]	58	SBP/DBP ≥210/105 mmHg in male, SBP/DBP ≥190/105 mmHg in female	56 ± 10	42	Bruce protocol	Case-control	In patients without resting hypertension, HRE is associated with slight systolic dysfunction
Sharabi et al. [34]	190	SBP/DBP ≥200/100 mmHg	42.6	100	Bruce protocol	Case-control	The risk of developing hypertension was higher in subjects with HRE than in the control group

*N* = 28′031, *x˜* age = 48.75, IQR = 10, *x˜* (% male) = 69, IQR = 44.1. Abbreviations: HRE, hypertensive response to exercise; LVMI, left ventricular mass index; CRP, C reactive protein; BMI, body mass index; DBP, diastolic blood pressure; ABPM, ambulatory blood pressure monitoring; BP, blood pressure; MH, masked hypertension; RAAS, renin-angiotensin-aldosterone system; SBP, systolic blood pressure; LV, left ventricular; NO, nitrogen monoxide; CV, cardiovascular; CVD, cardiovascular disease; GMP, guanosine monophosphate; ADMA, asymmetric dimethylarginine.

**Table 2:** Study characteristics of included studies with athletes.

Author (year)	Study size (*n*)	Definition for HRE	Age (years)	Gender (% male)	Exercise testing protocol	Type of athlete	Study design	Summary of findings
Currie et al. [35]	22	SBP 190–210 mmHg	55.5 ± 5	73	Graded exercise test	Endurance athletes	Cross-sectional	No cardiovascular dysfunction. Similar parameters of sympathetic reactivity, LV structure and function and central arterial stiffness were observed in athletes with and without HRE
Turmel et al. [36]	44	SBP ≥220 mmHg, DBP ≥110 mmHg	20	63.2	Progressive maximal aerobic exercise test (RAMP)	Endurance athletes	Cross-sectional	Athletes with HRE had a higher BP during exercise at every intensity and higher SBP during 24 h ABPM. Lower apo-A1 serum levels in athletes with HRE imply a higher risk for cardiovascular disease
Caselli et al. [13]	1876	SBP ≥220 mmHg for male, 200 mmHg for female, DBP ≥85 mmHg for male, ≥80 mmHg for female	25 ± 6	64	Symptom-limited bicycle testing; increase in workload of 0.5 W/kg every 2 min	Olympic athletes classified in 4 subgroups; skill, power, mixed, endurance	Cross-sectional	7.5% had HRE. These athletes had larger BMI and were more commonly engaged in endurance and mixed sports
Leischik et al. [37]	51	No definition	37	100	Progressive maximal aerobic exercise test (RAMP)	Triathletes	Cross-sectional	Athletes with HRE show higher LV mass than athletes without HRE
Malek et al. [38]	30	SBP ≥210 mmHg DBP ≥90 mmHg or increase of DBP more than 10 mmHg above resting values	40.9 ± 6.6	100	Exercise test on treadmill	Ultra-marathon runners	Cross-sectional	Athletes with high-normal BP showed higher interventricular septal thickness and higher left and right ventricular mass index. Combined with HRE these findings were more pronounced. Athletes with isolated HRE did not show LV hypertrophy
Tahir et al. [39]	83	SBP ≥214 mmHg	43 ± 10	65	Progressive maximal aerobic exercise test (RAMP); increase in workload of 20–40 W/min	Triathletes	Cross-sectional	In male triathletes, higher maximal SBP values during exercise and longer race distances in swimming and cycling were independent predictors for focal myocardial fibrosis
Tahir et al. [40]	30	No definition	45 ± 10	100	Post-race examinations	Triathletes	Cross-sectional	Triathletes with myocardial fibrosis had elevated post-race NT-proBNP levels, higher peak systolic HRE and lower post-race LA ejection fraction
Bauer et al. [41]	142	No definition	26 ± 5	100	Progressive maximal aerobic cycling ergometer test; increase in workload of 50 W every 2 min	Handball and ice hockey players	Cross-sectional	Athletes with the lowest SBP/MET slope had the lowest maximum SBP but achieved the highest absolute and relative workload
Caselli et al. [14]	141	SBP ≥220 mmHg for male, 200 mmHg for female, DBP ≥85 mmHg for male, ≥80 mmHg for female	26 ± 6	66	Symptom-limited bicycle testing; increase in workload of 0.5 W/kg every 2 min	Endurance, power and mixed disciplines	Prospective (6.5 ± 2.8)	Athletes with HRE had 3.6 times higher risk of developing hypertension compared to those with normal BP response to exercise. No cardiac remodeling during follow up was found
Kim et al. [42]	17	SBP ≥210 mmHg	51.7 ± 7.7	100	Bruce protocol	Amateur marathon runners	Case-control	Marathon runners with HRE showed a greater LVMI and had more LV diastolic dysfunction than hypertensive sedentary subjects

*N* = 2436, *x˜* age = 38.95, IQR = 19, *x˜* (% male) = 86.5, IQR 35. Abbreviations: HRE, hypertensive response to exercise; LVMI, left ventricular mass index; CRP, C reactive protein; BMI, body mass index; DBP, diastolic blood pressure; ABPM, ambulatory blood pressure monitoring; BP, blood pressure; MH, masked hypertension; RAAS, renin-angiotensin-aldosterone system; SBP, systolic blood pressure; LV, left ventricular; NO, nitrogen monoxide; CV, cardiovascular; CVD, cardiovascular disease; GMP, guanosine monophosphate; ADMA, asymmetric dimethylarginine.

## Supplementary Materials

Supplementary Materials Online supplement includes an expanded introduction about the pathophysiology of athletes' heart and an expanded discussion about the current evidence of the pathophysiology of HRE, as well as an expanded discussion about the current evidence of HRE as a precursor of cardiovascular diseases.
