# Supplementary material for: Hypertensive Response to Exercise in Athletes: Unremarkable Finding or Relevant Marker for Future Cardiovascular Complications?
Source: Int J Hypertens. 2022 Nov 1;2022:8476751. doi: 10.1155/2022/8476751 (PMC9678482; doi:10.1155/2022/8476751)
Supplement: Supplementary Materials — Online supplement includes an expanded introduction about the pathophysiology of athletes' heart and an expanded discussion about the current evidence of the pathophysiology of HRE, as well as an expanded discussion about the current evidence of HRE as a precursor of cardiovascular diseases. [file 8476751.f1.docx]

**Online supplement**

**Expanded introduction:**

Indeed, Pressler et al. found that athletes involved in dynamic sports showed higher systolic blood pressure (SBP) values due to higher afterloads, whereas static sports showed higher diastolic blood pressure (DBP) values as a result of increased peripheral resistance.^1^

Based on the Morganroth`s hypothesis, cardiac remodeling in athletes might depend on the different sport disciplines and their hemodynamic effects on the heart: as such, endurance training is characterized by a predominantly isotonic physiology which leads to eccentric left ventricular (LV) remodeling with chamber enlargement and wall thickening due to an increased volume load. On the other hand, isometric training is characterized by concentric LV reorganization without chamber enlargement because of increased systemic vessel resistance. Athletes engaged in mixed sports show variable stages of cardiac remodeling.^2^ However, the Morganroth hypothesis has been recently challenged. Haykowsky et al. revised the hypothesis that endurance and resistance exercise develop different patterns of cardiac remodeling. They suggested that the cardiac remodeling of an athlete depends on the quantity of hemodynamic stress exposure, defined as intensity and duration of training load, not depending on the specific sports discipline.^3,4^

Cardiac output and systolic blood pressure raise linearly with the increment of work load in order to cover the metabolic needs of the muscles under effort. The diastolic blood pressure remains physiologically stable or decreases minimally.^5^

**Expanded results:**

*Pathophysiology of HRE*

Different studies show possible pathophysiological mechanisms of HRE. Under physical stress, the sympathetic nervous system and the renin-angiotensin-aldosterone system (RAAS) play an important role. Shim et al. investigated the neuro-hormonal changes in subjects with and without HRE and found an increase in RAAS activation during exercise with significantly higher Angiotensin II levels.^6^ Similarly, Tzemos et al. found elevated Angiotensin II levels in subjects with HRE at peak exercise, showing a neurohormonal dysregulation in those patients. In subjects with HRE, the endothelial dysfunction was associated with a reduced nitric oxide (NO) activity, which consequently leads to an exaggerated response to vasoconstrictors.^7^ Chang et al. found an endothelial dysfunction in subject with HRE due to an impaired NO/cyclic guanosine monophosphate (cGMP) pathway. Those subjects showed a lower cGMP during exercise.^8^ Stewart et al. also showed a possible correlation between HRE and an endothelial vasodilator dysfunction in an observational study.^9^ Kayrak et al. demonstrated an increased serum asymmetric dimethylarginine (ADMA) level in subjects with HRE when compared to the control group. ADMA seems to inactivate NO, leading therefore to an impaired vasodilatory capacity. This impairment could be an explanation for HRE.^10^ Sharman et al. found an impaired neural baroreflex sensitivity in subjects with HRE independently from arterial carotid stiffness or other cardiovascular (CV) risk factors.^11^ Jae et al. found an association between high uric acid serum levels and HRE after adjusting for established cardiovascular risk factors, thus proposing as possible pathophysiological explanation that increased serum uric acid levels activates the RAAS and reduces the NO concentration, which in turn induces a vasoconstriction leading to HRE.^12^

Jae et al. showed that individuals with HRE had an increased risk of carotid atherosclerosis when compared to subjects without HRE even after adjusting for other risk factors.^13^ In another study Jae et al. found elevated levels of white blood cell in subjects with HRE and, therefore, speculate an inflammatory process as pathophysiological process for the development of hypertension.^14^ Papavasileiou et al. found an impaired glucose metabolism in nondiabetic patients with HRE and speculate that this may lead to future cardiovascular implications.^15^

Thanassoulis et al. found a correlation between classic cardiovascular risk factors, arterial stiffness, impaired endothelial function and HRE.^16^

**Expanded discussion:**

**HRE precedes hypertension and other cardiovascular complications**

We found 6 prospective studies that found a correlation between HRE and an increased risk of developing hypertension.

Schultz et al. performed a meta-analysis including 12 longitudinal studies for a total of 46,314 subjects. Cardiovascular events and mortality rates were documented during a mean follow-up of 15 years. Subjects with HRE at moderate exercise intensities had a 36% greater rate of cardiovascular events and mortality compared to subjects without HRE.^17^ Therefore, the detection of HRE may help in identifying subjects at higher risk for adverse cardiovascular events.

Some studies, however, go in the opposite direction. Ramos et al. found a reduced vagal tone in patients with HRE, thus suggesting a favorable clinical prognosis.^18^ In a population of elderly non-athletes with HRE, Hedberg et al. suggested an improved long-term survival.^19^ Lima et al. could not demonstrate a correlation between HRE and the development of resting hypertension. The only risk factors influencing new-onset hypertension were higher age and increased BMI.^20^ Such discrepancies may be due to the different characteristics of the study population, the methodology used for exercise testing and the definition for HRE. Leiba et al. compared two different definitions of HRE and its association to cardiorespiratory fitness. They showed that depending on the definition used, the cardiorespiratory fitness and cardiovascular risk profile change. The first definition was based on the absolute SBP threshold $\geq$ 200mmHg, the second one was corrected for the maximal workload achieved.^21^ As mentioned above, there is no universally accepted definition of HRE. Especially when trying to determine the prognostic cut-off values, there is inconsistency in the available literature. Jae et al. found that subjects with peak SBP higher than 181 mmHg and relative SBP increase (peak SBP minus resting SBP) greater than 52 mmHg during exercise had higher risk in developing hypertension. Hence, these levels may represent a predictive threshold for the development of future hypertension.^22^ Mariampillai et al. investigated if there is a threshold level to define HRE at moderate workload, which may lead to a greater risk of coronary heart disease (CHD). Their results showed a linear association between HRE at moderate workload and the risk of developing CHD, without determining a specific threshold level for CHD.^23^

Hedman et al. proposed a more physiologically suitable measure to identify subjects with HRE than using absolute peak SBP. After evaluating a workload-indexed blood pressure response (increase in SBP per increase in metabolic equivalent of task (SBP/MET slope)), they propose that SBP/MET slope could be used as a more specific parameter to identify subjects with HRE and found indeed a positive correlation with CV mortality. In contrast, in this study, peak SBP wasn’t associated with increased risk of mortality.^24^ This parameter may thus represent an additional marker to evaluate cardiovascular risk in apparently healthy individuals.

Since angiotensin II influences the vascular and myocardial response to exercise, angiotensin II receptor blocker (AT-I) or angiotensin converting enzyme inhibitor (ACE-I) could represent a first-line therapy in athletes with HRE. However, at the moment a pharmacological therapy is not indicated, unless a masked hypertension is discovered, as no evidence is yet available that antihypertensive treatment in HRE is in any way beneficial.^25^ It is important to recognize that these two drugs do not affect exercise capacity nor are they listed as doping, while other antihypertensives are considered as doping substances. For some skill sports like archery and shooting, beta-blockers for example, are considered as doping, while exercise performance might be negatively affected for endurance sports. Diuretics are forbidden in all kind of competitive sports.^26^ However, both AT-I and ACE-I are contraindicated during pregnancy and female athletes should be aware of this.

LV hypertrophy caused by endurance training is reversible after a detraining period. Differently, cardiac remodeling triggered by hypertension usually responds well to antihypertensive medication, however, LV hypertrophy may not be reverted anymore, thus representing a rational to treat athletes with HRE.^26^

In the EAPC recommendations, athletes with hypertension should have regular cardiovascular examinations such as electrocardiogram and BP evaluation both at rest and during exercise. Consequently, they are also checked for HRE. Special attention should be paid to athletes with associated other cardiovascular risk factors. Annual follow-ups are recommended. The decision to restrain athletes from competitions should be individualized and depend upon the comprehensive cardiovascular risk evaluation: subjects with high to very high cardiovascular risk, target organ damage or whenever BP values cannot be lowered to a normal range, should be temporary restricted from competitive sports, especially in power disciplines.^26^ Non-athletes with low or moderate cardiovascular risk are allowed to perform high-intensity exercise even if they develop high SBP levels during exercise.^26^

1. Pressler A, Jahnig A, Halle M, et al. Blood pressure response to maximal dynamic exercise testing in an athletic population. *J Hypertens* 2018;36(9):1803-09. doi: 10.1097/hjh.0000000000001791 [published Online First: 2018/05/26]

2. Hegde SM, Solomon SD. Influence of Physical Activity on Hypertension and Cardiac Structure and Function. *Curr Hypertens Rep* 2015;17(10):77. doi: 10.1007/s11906-015-0588-3 [published Online First: 2015/08/19]

3. Kooreman Z, Giraldeau G, Finocchiaro G, et al. Athletic Remodeling in Female College Athletes: The "Morganroth Hypothesis" Revisited. *Clin J Sport Med* 2019;29(3):224-31. doi: 10.1097/jsm.0000000000000501 [published Online First: 2019/04/30]

4. Haykowsky MJ, Samuel TJ, Nelson MD, et al. Athlete's Heart: Is the Morganroth Hypothesis Obsolete? *Heart Lung Circ* 2018;27(9):1037-41. doi: 10.1016/j.hlc.2018.04.289 [published Online First: 2018/05/19]

5. Currie KD, Floras JS, La Gerche A, et al. Exercise Blood Pressure Guidelines: Time to Re-evaluate What is Normal and Exaggerated? *Sports Med* 2018;48(8):1763-71. doi: 10.1007/s40279-018-0900-x [published Online First: 2018/03/27]

6. Shim CY, Ha JW, Park S, et al. Exaggerated blood pressure response to exercise is associated with augmented rise of angiotensin II during exercise. *J Am Coll Cardiol* 2008;52(4):287-92. doi: 10.1016/j.jacc.2008.03.052 [published Online First: 2008/07/19]

7. Tzemos N, Lim PO, Mackenzie IS, et al. Exaggerated Exercise Blood Pressure Response and Future Cardiovascular Disease. *J Clin Hypertens (Greenwich)* 2015;17(11):837-44. doi: 10.1111/jch.12629 [published Online First: 2015/08/04]

8. Chang HJ, Chung JH, Choi BJ, et al. Endothelial dysfunction and alteration of nitric oxide/ cyclic GMP pathway in patients with exercise-induced hypertension. *Yonsei Med J* 2003;44(6):1014-20. doi: 10.3349/ymj.2003.44.6.1014 [published Online First: 2004/01/03]

9. Stewart KJ, Sung J, Silber HA, et al. Exaggerated exercise blood pressure is related to impaired endothelial vasodilator function. *Am J Hypertens* 2004;17(4):314-20. doi: 10.1016/s0895-7061(03)01003-3 [published Online First: 2004/04/06]

10. Kayrak M, Bacaksiz A, Vatankulu MA, et al. Association between exaggerated blood pressure response to exercise and serum asymmetric dimethylarginine levels. *Circ J* 2010;74(6):1135-41. doi: 10.1253/circj.cj-09-0419 [published Online First: 2010/05/11]

11. Sharman JE, Boutouyrie P, Perier MC, et al. Impaired baroreflex sensitivity, carotid stiffness, and exaggerated exercise blood pressure: a community-based analysis from the Paris Prospective Study III. *Eur Heart J* 2018;39(7):599-606. doi: 10.1093/eurheartj/ehx714 [published Online First: 2017/12/28]

12. Jae SY, Bunsawat K, Choi YH, et al. Relation of serum uric acid to an exaggerated systolic blood pressure response to exercise testing in men with normotension. *J Clin Hypertens (Greenwich)* 2018;20(3):551-56. doi: 10.1111/jch.13219 [published Online First: 2018/02/20]

13. Jae SY, Fernhall B, Heffernan KS, et al. Exaggerated blood pressure response to exercise is associated with carotid atherosclerosis in apparently healthy men. *J Hypertens* 2006;24(5):881-7. doi: 10.1097/01.hjh.0000222758.54111.e2 [published Online First: 2006/04/14]

14. Jae SY, Fernhall B, Lee M, et al. Exaggerated blood pressure response to exercise is associated with inflammatory markers. *J Cardiopulm Rehabil* 2006;26(3):145-9. doi: 10.1097/00008483-200605000-00005 [published Online First: 2006/06/02]

15. Papavasileiou MV, Thomopoulos C, Antoniou I, et al. Impaired glucose metabolism and the exaggerated blood pressure response to exercise treadmill testing in normotensive patients. *J Clin Hypertens (Greenwich)* 2009;11(11):627-35. doi: 10.1111/j.1751-7176.2009.00172.x [published Online First: 2009/11/03]

16. Thanassoulis G, Lyass A, Benjamin EJ, et al. Relations of exercise blood pressure response to cardiovascular risk factors and vascular function in the Framingham Heart Study. *Circulation* 2012;125(23):2836-43. doi: 10.1161/circulationaha.111.063933 [published Online First: 2012/05/11]

17. Schultz MG, Otahal P, Cleland VJ, et al. Exercise-induced hypertension, cardiovascular events, and mortality in patients undergoing exercise stress testing: a systematic review and meta-analysis. *Am J Hypertens* 2013;26(3):357-66. doi: 10.1093/ajh/hps053 [published Online First: 2013/02/06]

18. Ramos PS, Araújo CG. Normotensive individuals with exaggerated exercise blood pressure response have increased cardiac vagal tone. *Arq Bras Cardiol* 2010;95(1):85-90. doi: 10.1590/s0066-782x2010005000078 [published Online First: 2010/06/22]

19. Hedberg P, Ohrvik J, Lönnberg I, et al. Augmented blood pressure response to exercise is associated with improved long-term survival in older people. *Heart* 2009;95(13):1072-8. doi: 10.1136/hrt.2008.162172 [published Online First: 2009/03/24]

20. Lima SG, Albuquerque MF, Oliveira JR, et al. Exaggerated blood pressure response during the exercise treadmill test as a risk factor for hypertension. *Braz J Med Biol Res* 2013;46(4):368-47. doi: 10.1590/1414-431x20132830 [published Online First: 2013/04/20]

21. Leiba A, Baur DM, Kales SN. Exercise-induced hypertension among healthy firefighters-a comparison between two different definitions. *J Am Soc Hypertens* 2013;7(1):40-5. doi: 10.1016/j.jash.2012.11.002 [published Online First: 2012/12/19]

22. Jae SY, Franklin BA, Choo J, et al. Exaggerated Exercise Blood Pressure Response During Treadmill Testing as a Predictor of Future Hypertension in Men: A Longitudinal Study. *Am J Hypertens* 2015;28(11):1362-7. doi: 10.1093/ajh/hpv036 [published Online First: 2015/04/01]

23. Mariampillai JE, Liestøl K, Kjeldsen SE, et al. Exercise Systolic Blood Pressure at Moderate Workload Is Linearly Associated With Coronary Disease Risk in Healthy Men. *Hypertension* 2020;75(1):44-50. doi: 10.1161/hypertensionaha.119.13528 [published Online First: 2019/11/19]

24. Hedman K, Cauwenberghs N, Christle JW, et al. Workload-indexed blood pressure response is superior to peak systolic blood pressure in predicting all-cause mortality. *Eur J Prev Cardiol* 2019:2047487319877268. doi: 10.1177/2047487319877268 [published Online First: 2019/10/01]

25. Kim D, Ha JW. Hypertensive response to exercise: mechanisms and clinical implication. *Clin Hypertens* 2016;22:17. doi: 10.1186/s40885-016-0052-y [published Online First: 2016/07/29]

26. Niebauer J, Borjesson M, Carre F, et al. Recommendations for participation in competitive sports of athletes with arterial hypertension: a position statement from the sports cardiology section of the European Association of Preventive Cardiology (EAPC). *Eur Heart J* 2018;39(40):3664-71. doi: 10.1093/eurheartj/ehy511 [published Online First: 2018/08/31]
